# Thoracoscopic-guided paraesophageal abscess drainage in foreign body-induced esophageal perforation: a case report

**DOI:** 10.1093/jscr/rjag189

**Published:** 2026-03-22

**Authors:** Mu-Chou Lin, Ying-Yuan Chen

**Affiliations:** Division of Thoracic Surgery, Department of Surgery, National Cheng Kung University Hospital, College of Medicine, National Cheng Kung University, 138 Shengli Rd., North Dist. Tainan 704302, Taiwan; Division of Thoracic Surgery, Department of Surgery, National Cheng Kung University Hospital, College of Medicine, National Cheng Kung University, 138 Shengli Rd., North Dist. Tainan 704302, Taiwan

**Keywords:** esophageal perforation, mediastinal abscess, percutaneous drainage, thoracoscopic surgery

## Abstract

Esophageal perforation is a life-threatening emergency, especially when diagnosis is delayed or when the thoracic esophagus is involved. Controlling mediastinal contamination is critical, yet the optimal drainage strategy for contained mediastinal abscesses remains controversial. We report the case of a 76-year-old woman with a foreign body-induced lower thoracic esophageal perforation complicated by a contained paraesophageal abscess. After endoscopic removal of an impacted blister pack, primary repair was not feasible due to severe inflammation and friable tissue. Video-assisted thoracoscopic exploration revealed an intact mediastinal pleura with a localized abscess. Thoracoscopically guided percutaneous drainage using a 14-Fr pigtail catheter was performed to achieve source control while avoiding uncontrolled pleural contamination. The patient recovered uneventfully, with complete healing of the esophageal perforation. This case demonstrates that thoracoscopically guided percutaneous drainage can be a safe, organ-preserving option for carefully selected patients with contained mediastinal abscesses.

## Introduction

Esophageal perforation is an uncommon but potentially life-threatening condition, particularly with delayed diagnosis or thoracic involvement. Mortality remains high and is largely determined by the extent of mediastinal or pleural contamination and adequacy of source control [1, 2]. Although early primary repair is considered standard, delayed presentation often necessitates alternative strategies focused on infection control and nutritional support [3, 4].

Fundamental management principles include prompt pleural and mediastinal drainage, eradication of infection with broad-spectrum antibiotics, and enteral nutritional support [2, 5]. While nonoperative management may be feasible in selected patients, operative intervention is frequently required to achieve reliable source control [3].

The optimal drainage strategy for localized mediastinal abscesses remains controversial. Conventional incision and drainage may disrupt an initially contained infection, whereas endoscopic or computed tomography (CT)-guided percutaneous drainage is limited by restricted visualization and procedural control [6, 7]. In patients with preserved mediastinal pleural integrity, thoracoscopically guided drainage may provide an organ-preserving alternative. We report a case of foreign body-induced lower thoracic esophageal perforation complicated by a contained paraesophageal abscess, successfully managed using this approach.

## Case report

A 76-year-old woman with a history of gastroesophageal reflux disease and gastric mucosa-associated lymphoid tissue lymphoma under regular surveillance presented to the emergency department with epigastric pain and progressive dysphagia over 2 days. On arrival, her vital signs were stable, including a temperature of 37.3°C, heart rate of 95 beats/min, respiratory rate of 20 breaths/min, and blood pressure of 127/71 mmHg.

**Figure 1:**
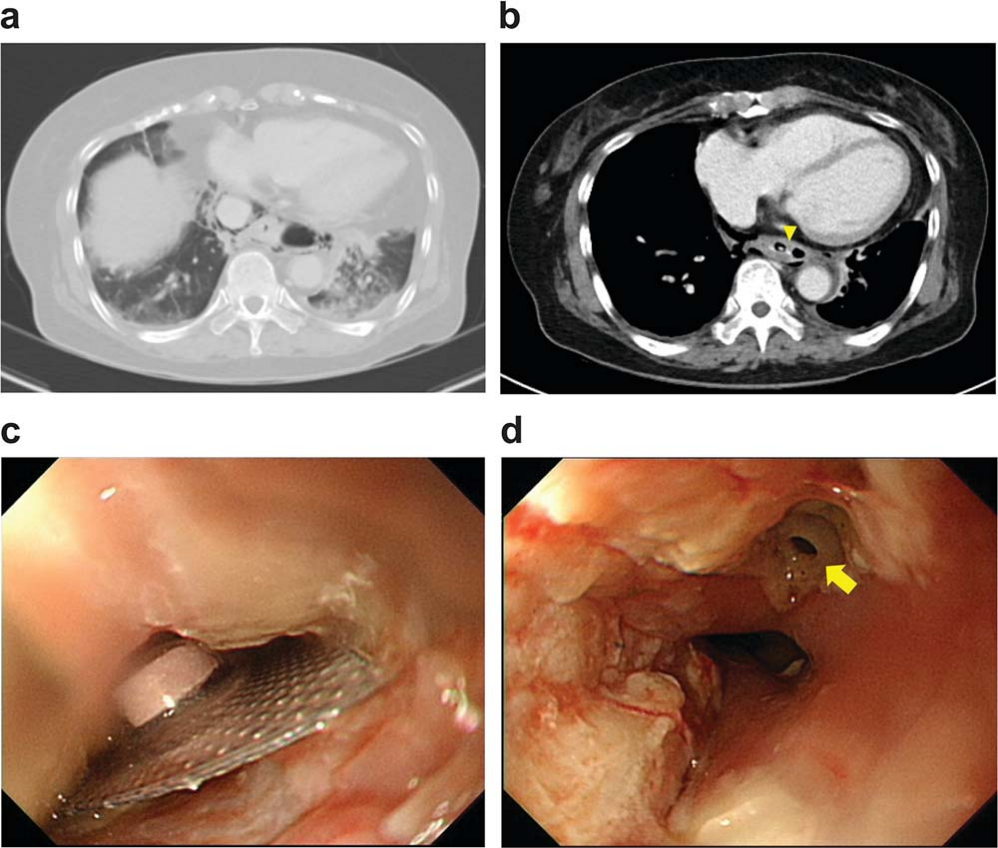
(a) Lung and (b) mediastinal windows show a radiopaque foreign body lodged in the lower thoracic esophagus (arrow tip). (c) Esophagogastroduodenoscopy reveals an impacted blister pack in the lower thoracic esophagus. (d) A 4 × 4 mm perforation visible after the blister was removed (solid arrow).

Upright chest radiography demonstrated a retrocardiac air pocket located between the T10 vertebral level and the descending aorta. Chest computed tomography (Fig. 1a and b) revealed a radiopaque foreign body lodged in the lower thoracic esophagus, approximately 3 cm above the esophagogastric junction, accompanied by pneumomediastinum and a small left pleural effusion. These findings raised strong concern for foreign body-induced esophageal perforation with mediastinal infection or early empyema. Following multidisciplinary consultation with the gastroenterology team, emergency surgical intervention under general anesthesia was undertaken.

Esophagogastroduodenoscopy identified an impacted blister pack in the lower thoracic esophagus (Fig. 1c), which was removed using endoscopic forceps. A 4 × 4 mm perforation with an acutely suppurative base was visualized at 35 cm from the incisors (Fig. 1d). Although the defect was small, the surrounding tissue was severely inflamed and friable, rendering primary or reinforced repair unfeasible. Given concern for mediastinal contamination, left-sided video-assisted thoracoscopic surgery (VATS) was performed for definitive evaluation and source control.

**Figure 2:**
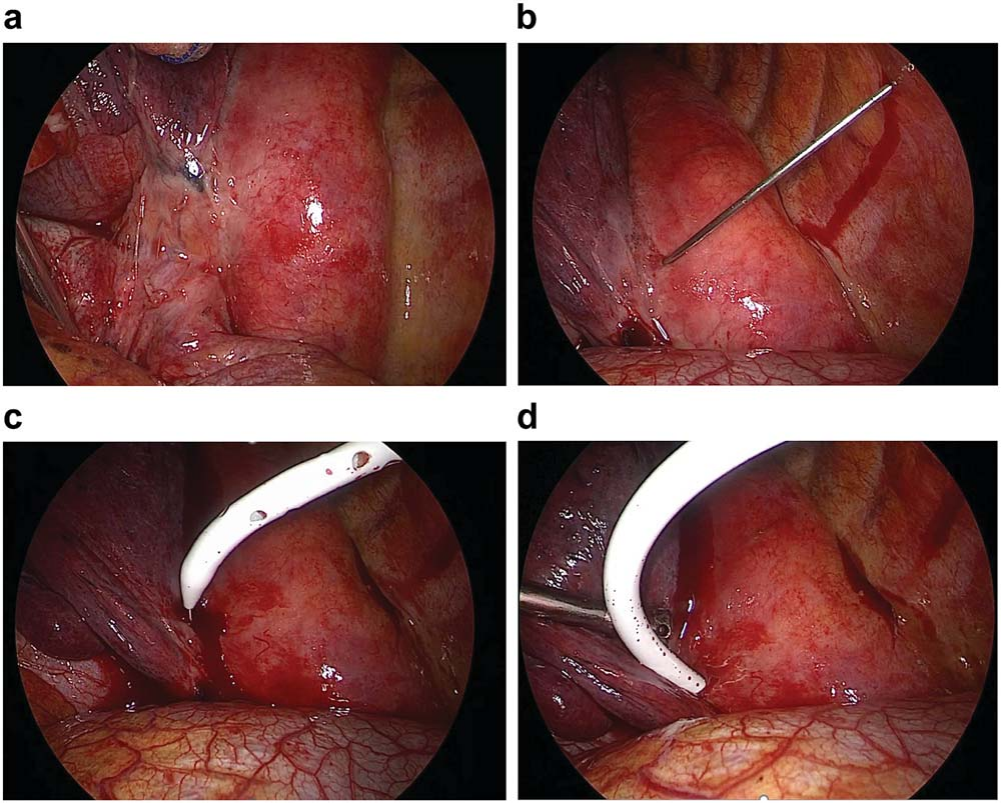
Video-assisted thoracoscopy. (a) Focal, tense bulging mass along the lower esophagus. (b–d) 14-Fr pigtail catheter introduced into the abscess cavity using the Seldinger technique.

Upon entry into the pleural cavity, a small volume of freely flowing turbid effusion was aspirated. Inspection of the posterior mediastinum revealed a focal, tense bulge along the left posteroinferior aspect of the esophagus (Fig. 2a), corresponding to the paraesophageal abscess identified on preoperative CT. The mediastinal pleura appeared intact, with no evidence of gross perforation or disruption.

To avoid creating a wide communication between the pleural space and the abscess cavity, a thoracoscopically guided percutaneous drainage strategy was chosen. Under direct thoracoscopic visualization, an 18-gauge Chiba needle was advanced into the bulging mediastinal lesion, yielding purulent material. A 14-Fr pigtail catheter (Cook Incorporated, Bloomington, USA) was then inserted using the Seldinger technique and advanced into the abscess cavity (Fig. 2b–d, Video 1), with continuous purulent return confirming correct placement. Residual purulent material was evacuated, and the pleural cavity was irrigated with 3000 ml of sterile normal saline until the effluent was clear.

Two 24-Fr chest tubes were placed before wound closure—one in the posterior costophrenic gutter and the other adjacent to the pigtail catheter—to facilitate postoperative drainage and monitoring. A feeding jejunostomy was constructed to ensure enteral nutrition.

**Figure 3:**
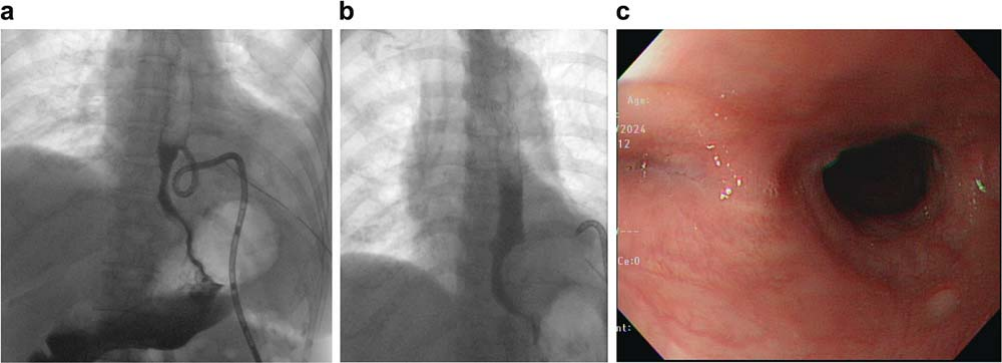
(a) Oral contrast esophagography on POD 30. (b) Oral contrast esophagography on POD 49. (c) Three-month follow-up endoscopy confirmed complete healing without stricture formation.

Postoperatively, the patient was kept nil per os, with continuous negative-pressure drainage via a vacuum reservoir connected to the pigtail catheter. Enteral feeding was initiated on postoperative day (POD) 3. Oral contrast esophagography on POD 30 (Fig. 3a) showed minimal contrast drainage through the pigtail catheter without extravasation, consistent with a controlled fistula. The catheter was gradually withdrawn. On POD 49, repeat imaging revealed a small residual blind-ending mediastinal pouch without evidence of leakage (Fig. 3b). The pigtail catheter was removed on POD 52. At 3-month follow-up, endoscopy confirmed complete healing without stricture formation (Fig. 3c), and the patient resumed a normal diet.

## Discussion

Management of esophageal perforation remains challenging, and therapeutic decisions should be individualized based on clinical status, imaging findings, and intraoperative assessment [2, 4]. Nonoperative management may be appropriate in selected patients with early-detected, instrument-related perforations, minimal symptoms, and well-contained mediastinal infection without distal obstruction [2, 3]. Adjunctive endoscopic clipping, stent placement, or endoluminal vacuum therapy have expanded options but remain limited by risks of migration, incomplete sealing, and reduced procedural control in contaminated fields [7].

In this case, endoscopic foreign body removal under general anesthesia allowed controlled extraction while minimizing the risk of exacerbating the esophageal defect. VATS provided direct visualization of the mediastinum and pleural cavity, enabling real-time assessment of contamination and selection of an appropriate drainage strategy. When infection remains localized, targeted drainage may suffice, whereas extensive mediastinal or pleural contamination necessitates more aggressive interventions, including wide debridement, pleural decortication, esophageal diversion, or T-tube placement. Given the anticipated need for prolonged fasting, enteral feeding access was essential to maintain nutritional status and avoiding long-term parenteral nutrition.

Thoracoscopic drainage of mediastinal abscesses secondary to esophageal perforation has been reported only sporadically. Chung and Ritchie described successful thoracoscopic drainage of a giant mediastinal abscess from cervical esophageal perforation using two 32-Fr chest tubes, resulting in survival of a patient with fulminant sepsis [8]. Subsequent reports have also documented favorable outcomes using thoracoscopic approaches in selected cases [9, 10]. In our case, a 14-Fr pigtail catheter was chosen to achieve controlled drainage. Compared with large-bore chest tubes, pigtail catheters are less prone to dislodgement, and Seldinger placement minimizes the risk of creating a large defect in the abscess wall. Prior CT-guided series have shown that small-caliber catheters can provide effective mediastinal abscess drainage [6].

Thoracoscopic-guided catheter placement offers additional advantages over CT-guided drainage, including avoidance of transgressing lung parenchyma and allow direct surgical control. When mediastinal pleural integrity is preserved and the abscess is well localized, this approach represents a feasible, organ-preserving option within a structured decision-making framework.

## Supplementary Material

Video_1_rjag189

## Data Availability

The datasets used and/or analyzed during the current study are available from the corresponding author on reasonable request.
